# Coronary artery bypass surgery and longitudinal evaluation of the autonomic cardiovascular function

**DOI:** 10.1186/cc3042

**Published:** 2005-01-26

**Authors:** Pedro Paulo S Soares, Adalgiza M Moreno, Sérgio LD Cravo, Antonio Claudio L Nóbrega

**Affiliations:** 1 Research Associate, Department of Physiology and Pharmacology, Universidade Federal Fluminense, Niterói, RJ, Brazil; 2 Physical Therapy Master Program, Centro Universitário do Triângulo Mineiro, Uberlândia, MG, Brazil; 3 Associate Professor, Department of Physiology, Universidade Federal de São Paulo, São Paulo, SP, Brazil; 4 Professor, Department of Physiology and Pharmacology, Universidade Federal Fluminense, Niterói, RJ, Brazil

## Abstract

**Introduction:**

Imbalance in autonomic cardiovascular function increases the risk for sudden death in patients with coronary artery disease (CAD), but the time course of the impact of coronary artery bypass grafting (CABG) on autonomic function has been little studied. Thus, the purpose of the present study was to determine the effects of the CABG on the cardiovascular autonomic function.

**Methods:**

Patients undergoing CABG (*n *= 13) and two matched control groups (patients with CAD who refused surgical treatment [*n *= 9], and healthy volunteers [*n *= 9]) underwent a prospective longitudinal study consisting of autonomic evaluation before and after (3, 6, 15, 30, 60, and 90 days) surgery, including measurement of heart rate variability (HRV), respiratory sinus arrhythmia (RSA), and Valsalva maneuver.

**Results:**

After CABG there was a decrease in, and a later recovery of, (1) the HRV in the time domain and in the frequency domain, (2) RSA, and (3) Valsalva maneuver.

**Conclusions:**

CABG caused an impairment, reversible after 60 days, of cardiovascular autonomic function, with a maximal decrease on about the sixth day after surgery.

## Introduction

Imbalance in autonomic cardiovascular function has been shown to increase the risk for ventricular arrhythmias and sudden death in patients with coronary artery disease (CAD) and after myocardial infarction [1,2]. Under these conditions there is an increased sympathetic adrenergic tone and reduced parasympathetic activity [3], a combination that causes augmented ventricular workload and oxygen demand, increasing the occurrence of ischemic events, and causes modification of the ionic currents across the cellular membrane, leading to direct electrical instability of myocytes.

The autonomic cardiovascular function has been traditionally evaluated by bedside tests such as respiratory sinus arrhythmia (RSA) and Valsalva maneuver, which measure the blood pressure and heart responses to standard stimuli [4,5]. In the past two decades, quantification of heart rate variability (HRV) has been used as an indicator of the autonomic control of sinus rate [6], providing independent predictive power for sudden death and all-cause mortality in CAD [1,2]. The signals necessary for HRV analysis can be obtained from electrocardiogram tracings recorded from short (15 min) resting periods to an entire day (24 hours) with multiple moments of physical activity. The HRV profile can be expressed both in the time domain, by measures of the variations in the R–R interval durations, and in the frequency domain, through spectral analysis [6].

Myocardial revascularization by coronary artery bypass grafting surgery (CABG) is an effective measure for reducing the symptoms and mortality in patients with unstable or severe CAD [7,8]. Despite the increasing importance of autonomic cardiac function for risk stratification in heart disease and, in contrast, the positive clinical outcome of CABG, there have been only few longitudinal studies investigating the impact of CABG on cardiac autonomic function. Although previous studies have indicated a diminished autonomic function after CABG [9-13], they could not describe properly the profile of cardiac autonomic function after surgery because they either lacked higher resolution in the first weeks after CABG or did not follow the patients up long enough to detect full recovery of cardiac autonomic indexes after CABG. The purpose of this study was therefore to evaluate, noninvasively and repeatedly, the cardiovascular autonomic function of patients with CAD during the initial three months after CABG, starting with a pre-surgery evaluation and repeated 3, 6, 15, 30, 60 and 90 days after surgery. Results obtained were compared with those from two control groups: matched patients with CAD but not undergoing CABG, and matched healthy volunteers.

## Methods

### Subjects

Thirteen patients (five female, eight male) undergoing CABG were recruited from the Procordis Hospital, Niterói, RJ, Brazil, fulfilling the following inclusion criteria: subjects of either sex, with diagnosed CAD (including laboratory tests, such as electrocardiogram, stress test, or coronary angiogram) and clinical indication for CABG. The exclusion criteria were as follows: presence of diabetes mellitus, congestive heart failure, previous cardiac surgery, recent (less than 6 months) myocardial infarction, implanted cardiac pacemaker, presence of atrial fibrillation, use of intraortic balloon, use of mechanical ventilation for more than 24 hours after surgery, myocardial infarction after surgery, and any other condition that could affect the autonomic function.

A control group (CAD; *n *= 9; five females, four males) for the surgical intervention was selected and paired by age and number of coronary vessels compromised (defined angiographically as more than 50% lumen obstruction). These patients also had clinical indication for CABG but refused to undergo surgery.

A further control group (healthy; *n *= 9; four females, five males) was included to evaluate the effect of CAD on autonomic function. The volunteers of this second control group were paired by age and the inclusion criteria for this group were to be asymptomatic and to have physiological responses to an exercise test without myocardial ischemia. The present study therefore employed two different groups as controls, one with patients with CABG who refused to undergo surgery and another consisting of healthy adults.

### Procedures

All subjects were informed with details about the protocol, which had been approved by the Institutional Review Board, and signed an informed consent form to participate in the study. They were instructed not to exercise or to take alcoholic or caffeinated drinks on the experimental days. All subjects were trained to perform autonomic tests with acceptable technical quality before actual measurements were made. Heart rate was recorded with a digital system for telemetry consisting of a transmitter placed on the subject's chest and a receptor and interface connected to a personal computer (Polar Vantage; Polar Electro Oy, Kempele, Finland). This system detects ventricular depolarization, corresponding to the R wave on the electrocardiogram, with a sampling rate of 500 Hz and a temporal resolution of 1 ms [14], and has been validated previously against standard Holter electrocardiography [15].

The patients in the CABG group were evaluated on the day preceding surgery and 3, 6, 15, 30, 60, and 90 days after surgery. The patients in the CAD group were tested four times in 3 months (0, 30, 60, and 90 days) and the volunteers in the healthy group were tested once.

### Respiratory sinus arrhythmia

After resting in the supine position for 15 min, the subjects were trained to perform respiratory cycles lasting 10–12 s, changing lung volume from maximal expiration (residual volume) to maximal inspiration (total lung capacity). The heart rate response to the RSA test was measured by the expiration/inspiration index (E/I index), calculated as the ratio between the longest R–R interval during expiration and the shortest R–R interval during inspiration.

### Valsalva maneuver

Shortly after resting for 15 min in sitting position, the subjects blew into a closed system connected to an aneroid manometer, exerting an expiratory pressure of 40 mmHg for 15 s, followed by expiration and spontaneous breathing. The Valsalva index was calculated by the ratio between the longest R–R interval after releasing the pressure and the shortest R–R interval during the forced expiratory pressure.

### Heart rate variability

The subjects rested for 10 min after the autonomic tests in the supine position and started a 15 min period of paced breathing at 18 cycles/min (0.30 Hz) and spontaneous tidal volume. The respiratory rate was kept constant with the aid of a metronome.

The original series of R–R intervals were filtered by a semi-automatic method [6], keeping the original time reference. The following parameters of HRV in the time domain were calculated: the standard deviation of all R–R intervals; the standard deviation of the averages of the R–R intervals calculated in 5 min segments; the percentage number of pairs of adjacent R–R intervals differing by more than 50 ms; and the square root of the mean of squares of differences between adjacent R–R intervals (RMSSD).

For frequency-domain analysis, the filtered R–R series were subjected to a cubic spline interpolation and decimated to be equally spaced in time. The decimated time series were used to calculate the power spectrum, calculated by the fast Fourier transform. The following parameters were analyzed: total power, power in the low-frequency (LF, 0.08–0.15 Hz) and high-frequency (HF, 0.15–0.40 Hz) bands, and the LF/HF ratio.

### Respiratory function

Forced expiratory vital capacity was measured (Spirodoc; Mir, Rome, Italy) before the autonomic tests. The expiratory maneuvers were performed after full inspiration (total lung capacity) in the seated position and repeated three times, taking the best result for analysis. Another series of forced expiratory maneuvers after full inspiration were used to measure the patient's peak expiratory pressure by manovacuometry (GEAR 07-01, Instrumentation Inc., Springfield, IL, USA). For this evaluation, the subjects were instructed to produce the effort with the chest and abdominal muscles and to keep the expiratory pressure for 3 s. The best result of three consecutive maneuvers was used for analysis.

### Statistical analysis

The patients' characteristics were compared by one-way analysis of variance (ANOVA) for the continuous variables or by Fisher's exact test for the proportions. Data from the autonomic evaluations were compared by a two-way ANOVA for repeated measures in which group (CABG, CAD, or healthy) and time were the main factors. The Bonferroni test was used for post-hoc pairwise analysis. Student's *t*-test was used to compare the results from patients with or without previous myocardial infarction within the CABG and CAD groups. The minimum necessary sample size was determined by setting the statistical power to 0.8 and the alpha error to 0.5. On the basis of the results of the R–R interval from pilot experiments and previous experience with patients undergoing CABG, we considered the minimum detectable difference to be 80 ms and the expected standard deviation of residuals as 50 ms for the ANOVA with three groups, yielding a sample size of nine subjects. All statistical procedures were performed on the SigmaStat^® ^(Jandel Scientific, San Rafael, CA, USA) and statistical significance was accepted at *P *< 0.05.

## Results

The results of the autonomic evaluation in patients with or without previous myocardial infarction in the CABG group (*n *= 5 and *n *= 8, respectively) and in the CAD group (*n *= 2 and *n *= 7, respectively) were similar. The data were therefore pooled within each group. The patients' characteristics are presented in Table 1. Note that gender proportion, age, body mass index and drugs used were not statistically different between groups.

### Respiratory sinus arrhythmia and Valsalva maneuver

**Figure 1:**
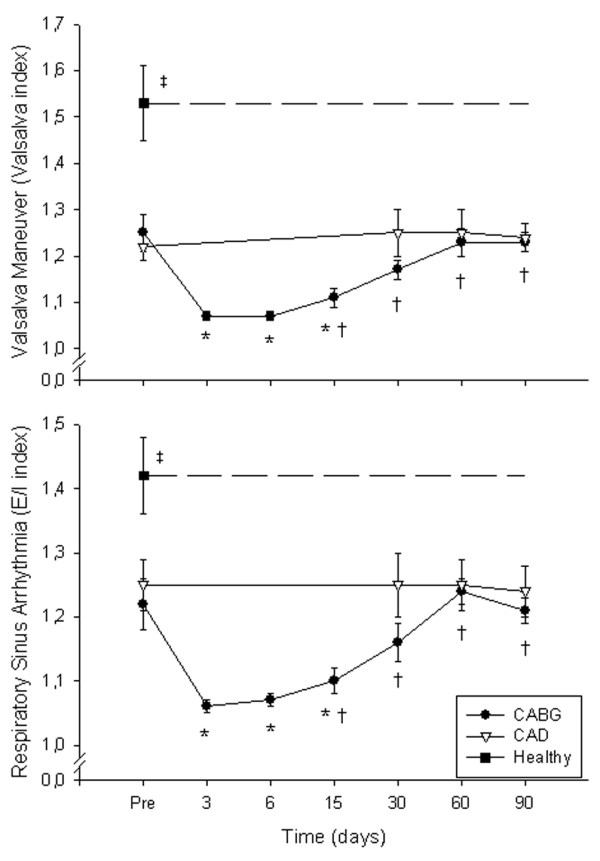
Longitudinal evaluation of autonomic cardiovascular tests in patients undergoing coronary artery bypass grafting surgery. Longitudinal autonomic cardiovascular evaluation (Valsalva maneuver and respiratory sinus arrhythmia) in the three groups: CABG, patients who underwent coronary artery bypass grafting; CAD, patients with coronary artery disease who did not undergo surgery; healthy, control subjects without CAD. *P < 0.05 versus CABG preoperative value; †*P *< 0.05 versus CABG day 3; ‡*P *< 0.05 versus CABG and CAD.

The indexes obtained during Valsalva maneuver and RSA presented a similar time profile in the CABG group. This profile was characterized by a marked decrease 3 days after surgery, followed by a significant increase after 15 days and a recovery to values similar to those observed before surgery after 30 days. Valsalva and RSA indexes in the CAD group were comparable to those observed in the CABG before surgery, and they did not change during the 90 days afterwards. Results for the healthy group were higher than those for the CABG and CAD groups (Fig. 1).

### Heart rate variability

The time-domain indexes of HRV decreased after CABG, returning to pre-surgery values by 30 or 60 days after surgery (Table 2). The values for the CAD group were similar to those from the CABG pre-surgery evaluation, whereas all values in the healthy group were higher than those for the CAD and CABG groups, except for the mean R–R interval and RMSSD.

**Figure 2:**
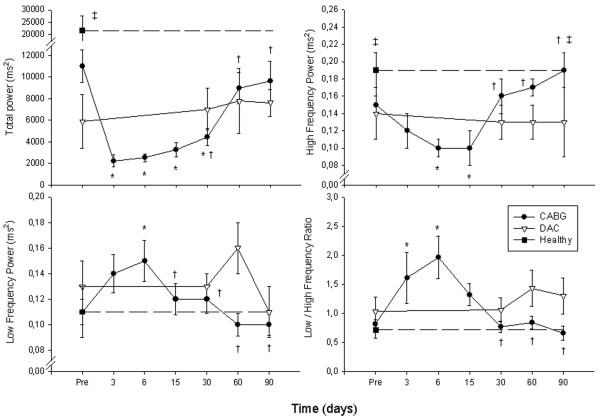
Longitudinal evaluation of heart rate variability in patients undergoing coronary artery bypass grafting surgery. Longitudinal results of spectral analysis of heart rate variability in the three groups: CABG, patients who underwent coronary artery bypass grafting; CAD, patients with coronary artery disease who did not undergo surgery; healthy, control subjects without CAD. Total power **(a) **and low-frequency power **(b) **indicate both adrenergic and parasympathetic modulation of heart rate; high-frequency power **(c) **represents the parasympathetic component; and the low-frequency/high-frequency ratio **(d) **represents autonomic balance that modulates heart rate. *P < 0.05 versus CABG preoperative value; †*P *< 0.05 versus CABG day 3 or 6; ‡*P *< 0.05 versus CABG and CAD, except high frequency, for which ‡*P *< 0.05 versus CAD.

Spectral analysis of HRV showed a similar change for total power when compared with the time-domain indexes; that is, a decrease in the first evaluation after CABG, a relative increase at 15 days, and a return to pre-surgery values at 60 days. The CAD and CABG groups presented similar values for this variable at the pre-surgery evaluation and after recovery of the CABG patients at 60 days. Total power for the healthy group was higher than those for the CAD and CABG groups (Fig. 2). HF power was similar in CABG and CAD patients before surgery, decreased after the intervention, and recovered after 30 days. LF power and the LF/HF ratio showed increases in the first days after surgery, followed by gradual decrements towards the pre-surgery values (Fig. 2). The results of the CAD group remained stable throughout the 90 days of evaluation.

### Respiratory function

Forced expiratory vital capacity and peak expiratory pressure were similar in the healthy, CAD and CABG groups before surgery and remained constant during the 90 days of follow-up in the CAD group. In the CABG group, both forced expiratory vital capacity and peak expiratory pressure decreased after surgery and returned to pre-intervention values by 15 days afterwards.

## Discussion

The present results have shown that CABG is followed by a depression of autonomic cardiac modulation, as demonstrated by reduced indices of conventional autonomic bedside tests and decreased HRV. The impaired autonomic modulation reached the lowest level 3–6 days after surgery, returning to pre-surgery values at about postoperative day (POD) 30–60. Although previous studies have shown similar trends [12,13], the present design employed an original approach, combining higher temporal resolution during the first month (PODs 3, 6, 15 and 31) with a follow-up long enough (POD 90) to detect the recovery of autonomic function to pre-surgery values.

Adequate control groups have been employed previously to evaluate the specific effect of CABG. For example, Hogue and colleagues [13] showed that patients undergoing CABG presented HRV indices 40–50% lower than patients undergoing nonthoracic vascular surgery, an effect that persisted for 5 days. Thus, the CABG itself reduced HRV. The work by Bronner and colleagues [11] employed patients undergoing aortic valve replacement as a specific control for patients undergoing cardiac surgery due to myocardial ischemia. These authors found that HRV decreased to similar proportions in the two groups, suggesting that the factors common to both surgical procedures, such as the cardiopulmonary bypass with cardioplegia and mechanical manipulation of the heart, were responsible for impairment of the autonomic function. In the present study, patients with CAD in similar clinical conditions to the CABG patients but who refused surgery were studied as a control for the surgical procedure group. As expected, the results from the autonomic tests and HRV variability before surgery were similar in the two groups and decreased after surgery, but did not change in the CAD group during 90 days. In addition, both groups have shown lower values of autonomic cardiac indexes in comparison with paired healthy subjects, emphasizing the effects of CAD on autonomic function. The study by Bauernschmitt and colleagues [9] also compared the results from patients undergoing CABG against those obtained from healthy volunteers, and showed that parasympathetic function was impaired 20 hours after surgery, but longer periods were not evaluated. The value of HRV as a tool for investigating autonomic function was also evaluated by Carpeggiani and colleagues [16], who showed that the LF spectral component has independent prognostic value early after acute myocardial infarction to predict in-hospital complications.

### Mechanisms of autonomic alterations in CABG

Various factors related to the CABG surgery could be involved in the impairment of cardiac autonomic modulation. Induction of anesthesia with fentanyl–diazepam–pancuronium have been demonstrated to decrease HRV, particularly the HF component, therefore increasing the LF/HF ratio [17]. These results suggest that this combination of drugs decreased vagal modulation and increased cardiac adrenergic activity (see below). In addition, Hogue and colleagues [13] have shown that induction of anesthesia before cardiopulmonary bypass surgery decreased HRV in comparison with the preoperative day. However, patients undergoing CABG presented a further decrease in HRV indices, revealing that the surgery had an effect besides that of anesthesia on autonomic function.

Several mechanisms have been suggested to impair autonomic function after CABG, such as mechanical destructions of autonomic fibers caused by aortic clamping [18,19], although an experiment in a canine model did not show a significant effect [20]. Extracorporal circulation could be theoretically involved as mechanism, but a recent study by Demirel and colleagues has not found a correlation between the duration of extracorporal circulation and the magnitude of autonomic function [12]. In addition, other factors involved with the surgical procedure, including cardioplegia and hypothermia, have been speculated to cause autonomic dysfunction [20] but have not been evaluated in a systematic manner. More recently, autonomic modulation of heart rate has been shown to be reduced by systemic inflammation [21], a condition known to occur after CABG [22].

### Clinical implications

The reduction of bedside tests and the time-domain indexes of HRV observed after CABG indicate an overall impairment in the autonomic modulation of heart rate. However, these indexes cannot distinguish between sympathetic adrenergic and parasympathetic function. Conversely, analysis of the power spectrum in the frequency domain has been used to provide insights into the relative contributions of the two branches of the autonomic nervous system to the global behavior of HRV [6]. The HF component is determined by vagal modulation, whereas the LF component carries the influence of both sympathetic adrenergic and parasympathetic modulation, and the ratio between LF and HF components should indicate sympathetic modulation, or the reciprocal relation of LF and HF components as a marker of the state of the sympathovagal balance [6]. In the present study, RSA, Valsalva maneuver, and time-domain indices of HRV showed patterns similar to those observed in the HF power; that is, nadir values at PODs 3–6 and recovery at about POD 30. It therefore seems that the HR response to the bedside tests and the time-domain indices of HRV have parasympathetic modulation as the major mechanism. In contrast, the LF component and the LF/HF ratio presented the exact opposite behavior, showing an increase after CABG with peak values at POD 6, returning to pre-surgery values at about POD 30. This should be interpreted as a reversible increase in the sympathetic drive after CABG and also suggests that, at least in these patients, the LF/HF ratio is linked mainly to adrenergic modulation.

The present results may have practical implications. Although a previous report by McHugh and colleagues did not find a relationship between HRV and cardiovascular instability in the intensive care unit after cardiac surgery [23], altered heart rate dynamics has been shown to be related to myocardial ischemic episodes in patents after CABG [23], suggesting that the autonomic nervous system has an important role in the pathogenesis of myocardial ischemia in the post-operative phase of CABG. Sympathetic activation increases cardiac oxygen demand, causing myocardial ischemia in patients with coronary obstruction. In addition, norepinephrine (noradrenaline) enhances myocardial electrical excitability and can trigger ectopic foci and ventricular arrhythmias [3]. In contrast, vagal stimulation decreases myocardial work and oxygen demand and is known to protect against cardiac arrhythmias acting on electrophysiological properties of the myocardium, such as increasing fibrillatory threshold [24,25]. Patients recovering from CABG might therefore be at augmented risk for myocardial ischemia and arrhythmias at about POD 3, when adrenergic influence is higher and vagal modulation is at its lowest. It is worth noticing that RSA and Valsalva maneuver, simple autonomic bedside tests, were able to show the impairment and recovery of autonomic function after CABG in a similar manner to that of HRV, which demands relatively more complex analytical methods. This might facilitate the application of autonomic evaluation after CABG in the clinical setting of a larger number of centers.

The results of the HF component are particularly interesting in the clinical perspective. The values for CABG patients were lower than those for healthy controls and quite similar to patients with CAD. After surgery there was a progressive increase in the HF values such that after 90 days these values were higher than those observed in CAD patients and identical to those of the healthy group. This suggests that CABG was able to induce an increase in cardiac vagal modulation in comparison with pre-surgery values, with potential protective action, because there is specific interest in measures capable of correcting parasympathetic dysfunction [26]. Similar results were obtained by Bellwon and colleagues [10]. Future studies should be conducted to establish whether autonomic evaluation is useful in predicting clinical outcome in patients after CABG and whether correction of vagal modulation can be considered as a mechanism for protection in patients undergoing CABG.

### Limitations of study

Because the present study involved only patients with normal ventricular function (ejection fraction more than 50%), we cannot extrapolate the results to other patients with poor ventricular function. In addition, the relatively small sample size, although sufficient to detect group differences, prevented us from exploring a potential difference between patients with coronary lesions affecting diverse ventricular walls. This would be an interesting aspect for exploration in future studies, because a more pronounced autonomic impairment has previously been shown in patients with myocardial ischemia on the anterior wall in comparison with those showing inferior wall ischemia [27]. Given that several pharmacological agents can interfere with autonomic function, the apparent more prevalent use of β-blockers in the CABG group could represent a confounding effect. Nevertheless, the proportion of drugs used was not statistically different, and the core analysis of the study was the longitudinal evaluation of autonomic function of each group, in which each subject served as his or her own control. Another limitation of the present study is that it cannot be determined which factors related to CABG were responsible for the autonomic changes (see above). Despite the underlying mechanism, it was quite clear that CABG and/or related factors caused reversible autonomic dysfunction with potential clinical impact.

## Conclusion

The results of this study show a decrease in the cardiovascular autonomic function indexes after CABG. The lowest values occurred 3–6 days after surgery with a gradual recovery, returning to preoperative values by POD 60. Future investigations should verify whether these results have practical implications for predicting clinical outcome; that is, whether the evaluation of autonomic function can detect patients with a higher risk after CABG.

## Key messages

• 	Cardiovascular autonomic function was impaired after CABG, as identified by noninvasive bedside tests.

• 	Autonomic function reached lowest values 3–6 days after CABG and returned to pre-surgery values after 60 days.

## Abbreviations

ANOVA = analysis of variance; CABG = coronary artery bypass grafting; CAD = coronary artery disease; HF = high-frequency; HRV = heart rate variability; LF = low-frequency; POD = postoperative day; RMSSD = square root of the mean of squares of differences between adjacent R–R intervals; RSA = respiratory sinus arrhythmia.

## Competing interests

The author(s) declare that they have no competing interests.

## Authors' contributions

ACLN conceived and designed the study, assisted with analysis and revised the manuscript. PPSS conducted the principal analysis and revised the manuscript. AMM participated in the study design, conducted data collection, assisted with analysis and drafted the manuscript. SLDC contributed to study design and manuscript revision. All authors read and approved the final manuscript.

## Figures and Tables

**Table 1:** Demographic characteristics of patients and control subjects

Characteristic	CABG (*n *= 13)	CAD (*n *= 9)	Healthy (*n *= 9)
Sex (males/females)	8/5	5/4	4/5
Age (years)	64 ± 2	64 ± 2	63 ± 2
Body mass index (kg/m^2^)	27.6 ± 0.6	27.4 ± 0.7	26.5 ± 0.9
Drug used			
ACE inhibitors/AngioII-blockers	5	6	-
Digital	3	0	-
Anti-arrhythmic	1	0	-
β-blockers	6	1	-
Calcium-channel antagonists	4	1	-
AAS	7	3	-

Values are means ± SEM. AAS, acetylsalicylic acid; ACE, angiotensin-converting enzyme; CABG, patients who underwent coronary artery bypass grafting; CAD, patients with coronary artery disease who did not undergo surgery; healthy, control subjects without CAD. The three groups were similar with regard to all variables (*P *> 0.05).

**Table 2:** Time-domain indexes of heart rate variability before and at various intervals after coronary artery bypass grafting

Variable	Group	Preoperative	After surgery					
			
			3 days	6 days	15 days	30 days	60 days	90 days
Mean R–R (ms)	CABG	903 ± 40	761 ± 45*	781 ± 42*	790 ± 36*	802 ± 26*	801 ± 36	802 ± 35
	CAD	856 ± 49	-	-	-	842 ± 38	816 ± 33	835 ± 47
	Healthy	860 ± 36	-	-	-	-	-	-
SDNN (ms)	CABG	35 ± 3	16 ± 2*	16 ± 2*	19 ± 2*	25 ± 2*	32 ± 3	27 ± 3
	CAD	31 ± 4	-	-	-	29 ± 3	30 ± 3	31 ± 2
	Healthy	42 ± 5^†^	-	-	-	-	-	-
SDANN (ms)	CABG	16 ± 3	6 ± 1*	6 ± 1*	9 ± 1*	15 ± 3	18 ± 3	14 ± 2
	CAD	21 ± 4	-	-	-	19 ± 3	20 ± 3	20 ± 3
	Healthy	39 ± 7^†^	-	-	-	-	-	-
RMSSD (ms)	CABG	24 ± 4	10 ± 2*	10 ± 1*	12 ± 1*	19 ± 4	21 ± 3	19 ± 2
	CAD	22 ± 3	-	-	-	18 ± 3	19 ± 2	20 ± 4
	Healthy	27 ± 3	-	-	-	-	-	-
pNN50 (%)	CABG	8 ± 0	3 ± 0*	3 ± 0*	3 ± 0*	4 ± 0*	7 ± 0	6 ± 0
	CAD	4 ± 0	-	-	-	3 ± 0	4 ± 0	3 ± 0
	Healthy	11 ± 0^b^	-	-	-	-	-	-

Values are means ± SEM. *P < 0.05 versus CABG preoperative value; ^†^*P *< 0.05 versus CABG and CAD. CABG, patients who underwent coronary artery bypass grafting; CAD, patients with coronary artery disease who did not undergo surgery; healthy, control subjects without CAD; pNN50, percentage number of pairs of adjacent R–R intervals differing by more than 50 ms; RMSSD, square root of the mean of squares of differences between adjacent R–R intervals; SDANN, standard deviation of the averages of the R–R intervals calculated in 5-minute segments; SDNN, standard deviation of all R–R intervals.
